# Effects of two measures of riparian plant biodiversity on litter decomposition and associated processes in stream microcosms

**DOI:** 10.1038/s41598-020-76656-4

**Published:** 2020-11-12

**Authors:** Naiara López-Rojo, Javier Pérez, Ana Basaguren, Jesús Pozo, Juan Rubio-Ríos, J. Jesús Casas, Luz Boyero

**Affiliations:** 1 Department of Plant Biology and Ecology, Faculty of Science and Technology, University of the Basque Country (UPV/EHU), 48940 Leioa, Spain; 2 Department of Biology and Geology, University of Almeria (UAL), 04120 Almería, Spain; 3 Andalusian Centre for the Evaluation and Monitoring of Global Change, CAESCG, Almería, Spain; 4 IKERBASQUE, Basque Foundation for Science, Bilbao, Spain

**Keywords:** Biodiversity, Freshwater ecology

## Abstract

Plant litter decomposition is a key ecosystem process that can be altered by global changes such as biodiversity loss. These effects can be particularly important in detritus-based ecosystems, such as headwater streams, which are mainly fuelled by allochthonous plant litter inputs. However, experiments examining effects of plant diversity on litter decomposition in streams have not reached consensus about which measures of biodiversity are more relevant. We explored the influence of two of these measures, plant species richness (SR; monocultures vs. 3-species mixtures) and phylogenetic distance (PD; species belonging to the same family vs. different families), on leaf litter decomposition and associated processes and variables (nutrient dynamics, fungal biomass and detritivore growth), in a stream microcosm experiment using litter from 9 tree species belonging to 3 families. We found a negative effect of SR on decomposition (which contradicted the results of previous experiments) but a positive effect on fungal biomass. While PD did not affect decomposition, both SR and PD altered nutrient dynamics: there was greater litter and detritivore N loss in low-PD mixtures, and greater litter P loss and detritivore P gain in monocultures. This suggested that the number of species in mixtures and the similarity of their traits both modulated nutrient availability and utilization by detritivores. Moreover, the greater fungal biomass with higher SR could imply positive effects on detritivores in the longer term. Our results provide new insights of the functional repercussions of biodiversity loss by going beyond the often-explored relationship between SR and decomposition, and reveal an influence of plant species phylogenetic relatedness on nutrient cycling that merits further investigation.

## Introduction

Current rates of biodiversity loss are far greater than those before human dominance of Earth^1–3^, as a result of multiple environmental changes of anthropogenic origin such as land transformation, climate change and species invasions^4–7^. Biodiversity loss, in turn, can alter ecosystem processes such as plant litter decomposition, which is key for the functioning of ecosystems^8^. Headwater streams are detritus-based ecosystems that are fuelled by allochthonous plant litter detritus inputs from the surrounding terrestrial catchment^9–11^. Once in the stream, plant litter is decomposed by microorganisms (mainly fungi) and invertebrates (litter-consuming detritivores), which involves the cycling of major nutrients such as nitrogen (N) and phosphorus (P), and the production of microbial and invertebrate biomass^12^. All these stream processes can be altered by multiple global environmental drivers (e.g., climate warming, eutrophication) and by terrestrial plant diversity loss, which is caused by widespread forestry practices such as monospecific plantations^13^.

There is evidence that plant diversity loss affects litter decomposition^14^, nutrient cycling^15^ and biomass production^16^, with effects mediated by complementary resource by detritivores (i.e., complementarity effects) or by the presence of particular litter types that decompose faster or slower than others (i.e., selection effects)^17^. However, inconsistencies between field and laboratory studies and across experiments^18^ suggest that there are still important gaps within this research field. A key question is whether species richness (SR; which has been used in most relevant studies) is the most appropriate measure of biodiversity, compared to other measures that consider the diversity of species traits^19,20^. Trait-related biodiversity measures could be expected to have greater influence on ecosystem processes than SR, because traits have direct functional repercussions^21^. For example, phylogenetic distance (PD) is often a good predictor of species trait variation^22–25^, and it has shown relationships with ecosystem processes such as primary production^26^ and litter decomposition^27^.

We experimentally explored how both plant SR and PD within litter assemblages influenced litter decomposition and associated processes and variables (nutrient dynamics, fungal biomass and detritivore growth) in stream microcosms. We examined the net diversity effect (i.e., the deviation between observed decomposition values in litter assemblages and the values expected from the corresponding monocultures) and, when possible, partitioned this effect into complementarity and selection effects^17^. We used leaf litter from 9 tree species belonging to 3 families (Betulaceae, Salicaceae and Fagaceae), which were introduced in microcosms (with and without detritivores) as monocultures (SR = 1) or mixtures (SR = 3) with either low PD (3 species from the same family) or high PD (3 species from 3 different families). The above processes were quantified after 6 weeks, and the following hypotheses were examined: (1) plant SR enhances all studied processes (i.e., they have greater values in mixtures than in monocultures)^15,28^, mostly due to complementarity effects^29^; (2) the difference between monocultures and mixtures is greater for high-PD than for low-PD mixtures; and (3) all the above patterns are more marked in the presence of detritivores, which often are key drivers of biodiversity-ecosystem process relationships^30–32^.

## Results

**Figure 1:**
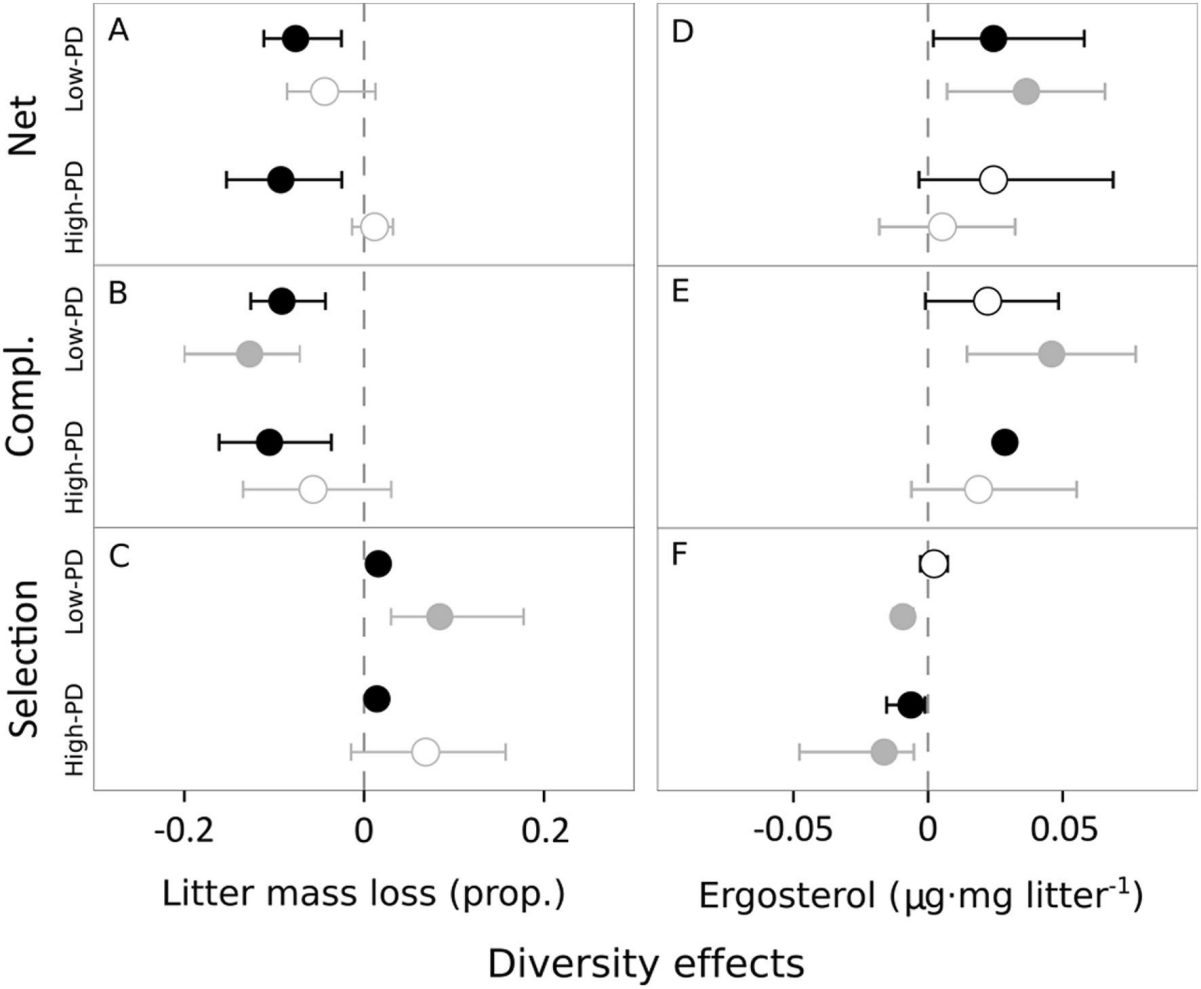
Net diversity, complementarity and selection effects for litter mass loss (proportion) and ergosterol content (µg mg litter^−1^) for low-PD and high-PD treatments, with (black) and without (grey) detritivores. Circles are means and whiskers denote upper and lower bounds of 95% nonparametric bootstrapped confidence intervals. Closed circles represent intervals that reject the null hypothesis (i.e., do not contain the value of zero) and open circles represent intervals that do not reject the null hypothesis.

Net diversity effects on decomposition (quantified through litter mass loss, LML) and fungal biomass (quantified through lipid ergosterol^33^) were not significantly affected by PD, either in microcosms with or without detritivores (Table 1, Table S3). The net diversity effect on decomposition was mostly negative (i.e., LML was higher in monocultures than in mixtures), and significant only in the presence of detritivores (Fig. 1A). In contrast, the net diversity effect on fungal biomass was positive (i.e., there was more ergosterol in mixtures than in monocultures), and the effect was significant only for low-PD mixtures (Fig. 1D). When net diversity effects on decomposition and fungal biomass were partitioned into complementarity and selection effects, there were again no differences between of PD treatments on any of the two variables (Table 1, Table S3), but they again showed different patterns: for decomposition, complementarity was negative (Fig. 1B) and selection was positive in low-PD (with and without detritivores) and high-PD mixtures (with detritivores: Fig. 1C); for fungal biomass, there was positive complementarity (significant in low-PD mixtures without detritivores and high-PD mixtures with detritivores; Fig. 1E) and negative selection (except for low-PD mixtures with detritivores; Fig. 1F). Diversity effects were thus almost entirely driven by complementarity effects in the presence of detritivores, with important contribution of selection effects in their absence (Table S4).

**Table 1:** Results of linear mixed-effects models testing for the effect of diversity, detritivore presence and their interaction on the response variables. Diversity levels were low-PD and high-PD treatments for net, complementarity and selection effects on decomposition (measured through leaf mass loss, LML) and fungal biomass (measured through ergosterol); and monocultures versus low-PD versus high-PD treatments for the change in litter and detritivore nitrogen (N) and phosphorus (P) and detritivore growth.

Variable	Effect	df	F	*p*
Net (LML)	Diversity	1,4	0.256	0.639
	Detritivore presence	1,49	3.340	0.073
	Diversity: Detr. presence	1,49	0.086	0.769
Complementarity (LML)	Diversity	1,4	0.040	0.854
	Detritivore presence	1,49	3.110	0.084
	Diversity: Detr. presence	1,49	0.080	0.777
Selection (LML)	Diversity	1,4	0.364	0.578
	Detritivore presence	1,49	0.042	0.837
	Diversity: Detr. presence	1,49	1.110	0.297
Net (Ergosterol)	Diversity	1,4	0.496	0.519
	Detritivore presence	1,28	0.021	0.884
	Diversity: Detr. presence	1,28	1.086	0.306
Complementarity (Ergosterol)	Diversity	1,4	0.860	0.406
	Detritivore presence	1,28	0.095	0.759
	Diversity: Detr. presence	1,28	0.144	0.706
Selection (Ergosterol)	Diversity	1,4	1.098	0.353
	Detritivore presence	1,28	5.298	0.029
	Diversity: Detr. presence	1,28	0.015	0.900
Change in litter N	Diversity	2,12	1.985	0.179
	Detritivore presence	1,13	0.658	0.418
	Diversity: Detr. presence	2,13	0.749	0.474
Change in litter P	Diversity	2,12	1.353	0.295
	Detritivore presence	1,13	4.955	0.027
	Diversity: Detr. presence	2,13	0.068	0.933
Detritivore growth	Diversity	2,12	0.049	0.951
Change in detritivore N	Diversity	2,12	14.538	< 0.001
Change in detritivore P	Diversity	2,12	1.429	0.279

*df* numerator and denominator degrees of freedom, *F* F-statistic value, *p* *p* value.

**Figure 2:**
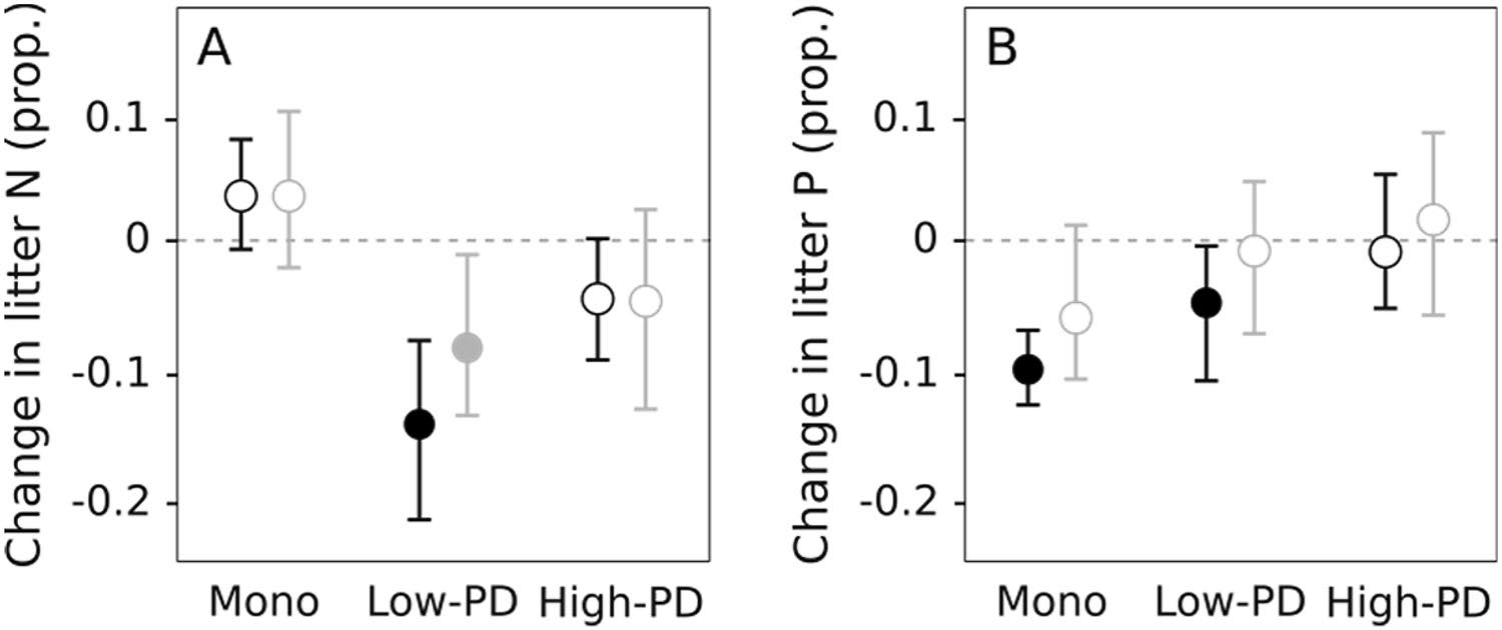
Proportional change in litter nitrogen and phosphorus content (mg g litter^−1^) for monocultures (Mono), low-PD and high-PD litter mixtures, with (black) and without (grey) detritivores. Circles represent means and whiskers denote upper and lower bounds of 95% nonparametric bootstrapped confidence intervals. Closed circles represent intervals that reject the null hypothesis (i.e., do not contain the value of zero) and open circles represent intervals that do not reject the null hypothesis.

**Figure 3:**
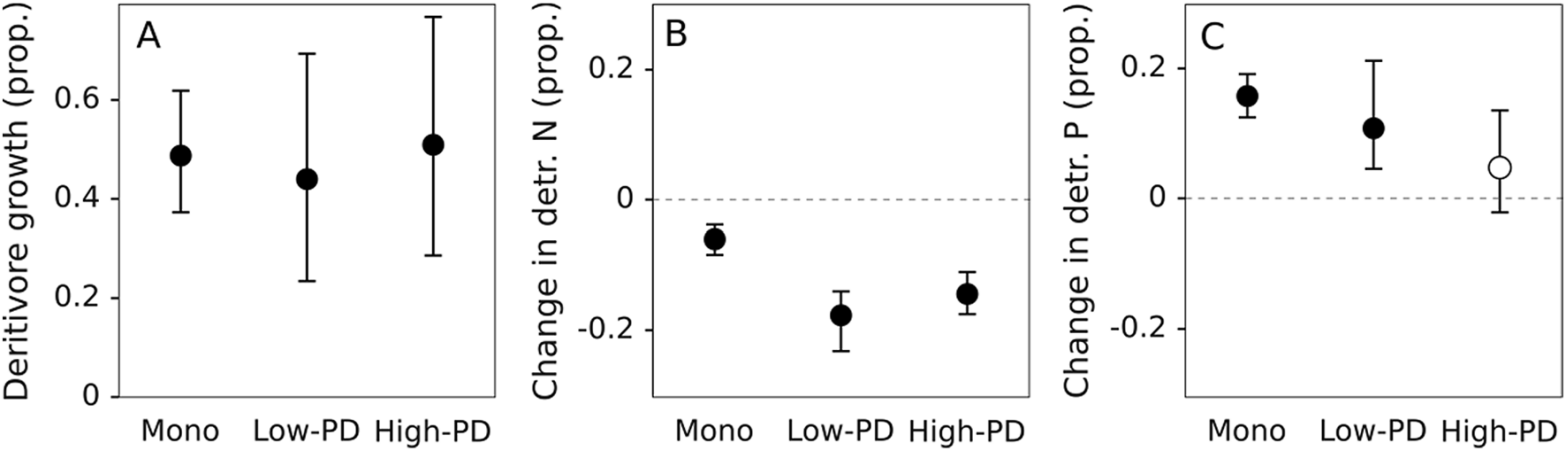
Detritivore growth and change in nitrogen and phosphorus (proportion) for monocultures (Mono), low-PD and high-PD litter mixtures. Circles represent means and whiskers denote upper and lower bounds of 95% nonparametric bootstrapped confidence intervals. Closed circles represent intervals that reject the null hypothesis (i.e., do not contain the value of zero) and open circles represent intervals that do not reject the null hypothesis.

Nutrient dynamics in litter showed differences between monocultures and mixtures, albeit not significant (Table 1, Table S3). Litter N concentration tended to increase in monocultures and decrease in mixtures, although the change in litter N was only significant for low-PD mixtures in the presence of detritivores (Fig. 2A). Litter P concentration decreased in the presence of detritivores in monocultures and low-PD mixtures, with no change in high-PD mixtures; and there was an increasing trend from monocultures to high-PD mixtures both in the presence and absence of detritivores (Fig. 2B). Detritivore growth was highly variable and showed no differences between treatments (Fig. 3A, Table 1). Detritivores decreased their N proportional content, and the decrease was higher in mixtures than in monocultures (Fig. 3B, Table 1). In contrast, detritivores increased their P proportional content in monocultures and low-PD mixtures and showed a similar but nonsignificant trend in high-PD mixtures (Fig. 3C, Table 1); the pattern shown was opposite to that in P litter concentration.

## Discussion

### Litter decomposition was lower in mixtures than in monocultures due to negative complementarity

Our experiment revealed a negative effect of plant species richness on litter decomposition: monocultures decomposed, on average, faster than litter mixtures. This result was unexpected when compared with several other microcosm experiments, which have found faster decomposition of litter mixtures than monocultures^15,28,30,31,34^. In most of the above-mentioned microcosm experiments, diversity effects occurred only in the presence of detritivores, suggesting that they were the key drivers of such effects, and the main underlying mechanism was a positive complementarity effect. Similarly, in our study, the diversity effect was significant in the presence of detritivores; in their absence, complementarity and selection effects presented similar but opposite values that counterbalanced each other (see below).

Positive complementarity can occur when different litter types offer complementary resources to consumers, or when the presence of one litter type enhances the consumption of another (i.e., facilitation), and is often greater than the positive selection effect (i.e., when a given litter type is decomposed faster than others). For example, an experiment found that complementarity accounted for 66% of the diversity effect on decomposition on average (and up to 99%) in several litter mixtures^30^. In our study, we also found that complementarity was the dominant mechanism behind diversity effects on decomposition in the presence of detritivores (selection effects were significant and positive, but only accounted for 13% of the net diversity effect on average) but, in this case, it was negative complementarity.

Negative complementarity was also found in a field study with a similar design to ours^25^, and could indicate some kind of physical or chemical interference between litter types. For example, toxic compounds present in one species could inhibit the consumption of another that would otherwise be consumed faster^35,36^. In our study, in the absence of detritivores, negative complementarity and positive selection were similar in magnitude (53% and 47% on average, respectively), resulting in a non-significant net diversity effect. This suggests that selection effects were more relevant for microbial than for detritivore-mediated decomposition, and indicates that the lack of net diversity effects on microbial decomposition found here and in other studies^15,30^ could be due to different mechanisms operating in opposite ways, rather than to the absence of interactions between litter diversity and microbial decomposers.

### Fungal biomass was higher in litter mixtures, mostly in those with low phylogenetic distance

Despite the negative effect of plant species richness on decomposition, the effect on fungal biomass was opposite, that is, litter mixtures produced more fungal biomass than expected from monocultures. This may result in greater litter conditioning^37^ and thus enhance detritivore-mediated decomposition in the longer term. However, this was significant only for low-PD mixtures (with and without detritivores), and driven by positive complementarity (which accounted for 87% of the net diversity effect on average), suggesting the existence of resource partitioning or facilitation among fungal species. This can occur if different species within the fungal assemblage differ in their enzymatic complements or activity patterns^18^, or benefit from the presence of litter types differing in physical structure [e.g., contrasting toughness or specific leaf area (SLA)], which increase habitat complexity and stability. However, we cannot confirm this as we did not characterize fungal assemblages. Moreover, in our case, such effects did not translate into differences in microbial decomposition, such as those shown in terrestrial ecosystems^38^, possibly due to functional redundancy of fungal species^18^.

Although we did not quantify fungal species richness, other studies have found that it is positively related to plant (litter) species richness, in relation to a higher functional trait diversity^39,40^. In our study, high-PD mixtures tended to have higher trait diversity than low-PD mixtures. Thus, it is possible that fungal assemblages growing on our high-PD mixtures were more diverse than those growing on low-PD mixtures, and more diverse fungal assemblages generally show slower production due to increased interspecific competitive interactions^18^. In high-PD assemblages, positive complementarity was the dominant mechanism (82% of the net diversity effect) only in the presence of detritivores, which most likely mediated this complementarity effect. In the absence of detritivores, positive complementarity and negative selection were similar in magnitude (53% and 47%, respectively), as occurred for decomposition, resulting in a very low and non-significant net diversity effect.

### Nutrient dynamics was influenced by plant species richness, with a lower influence of phylogenetic distance

Plant species richness affected the dynamics of N and P in litter and detritivores, but had no effect on detritivore growth, which was 42% on average (i.e., 1.14% per day); this is within the range reported elsewhere for *Sericostoma* spp. (0.75–2.99%)^15,41^. While litter monocultures tended to present higher N concentration (although the trend was not significant), it tended to be lower in mixtures (being the reduction significant only for low-PD mixtures, and significantly different from that of monocultures only in the presence of detritivores). This suggests that more N was used from litter in mixtures, which is in accordance with their higher fungal biomass, and with the key role of microorganisms in N dynamics shown elsewhere^15,31^. We note, however, that N litter content cannot be separated from N content of colonising fungi. In contrast, detritivores reduced their N proportional content in all cases, but less so when exposed to monocultures, suggesting that detritivores were able to use more N from litter when fungal biomass was lower. This counterintuitive pattern could be due to the fact that detritivores preferentially consume fungal biomass rather than the litter (i.e., the peanut butter instead of the cracker^42^), so their higher consumption could lead to higher N gain (or lower N loss in this case) in detritivores and lower fungal biomass at the same time. The general reduction in detritivore N content could be due to the fact no litter type fulfilled their N demands (even if *A. glutinosa* had high N concentration; Table S1); these demands are usually high for caddisflies because they use it for the production of silk and N-rich chitin for the exoskeleton^43^.

The dynamics of P showed a different pattern, which was opposite in litter and detritivores: litter decreased its P proportional content in monocultures and low-PD mixtures in the presence of detritivores, and detritivores increased their P proportional content when exposed to monocultures and low-PD mixtures; the trend was similar for high-PD mixtures in both cases, albeit not significant. This suggests that P dynamics were highly dependent on detritivores, which used P from monocultures and low-PD mixtures more efficiently than from high-PD mixtures, that is, from litter with lower diversity of functional traits in general or P in particular (P variability was 0.17% ± 0.09 SE in low-PD mixtures and 0.84% ± 0.39 in high-PD mixtures; Supplementary Table S2). This agrees with studies suggesting that detritivores can benefit from the concentration of resources^44,45^ and with the fact that detritivore growth was not constrained by P supply, as shown elsewhere^43^.

**Table 2:** Species comprising low-PD and high-PD litter mixtures (i.e., 3 plant species from the same family, or 3 species each from a different family, respectively), and trait variability (measured through RaoQ; value for each mixture and mean ± SE for each mixture type); PD: phylogenetic distance.

Litter mixtures	RaoQ
**Low-PD**	1.84 ± 0.46
*Alnus glutinosa* + *Betula celtiberica* + *Corylus avellana*	2.74
*Populus nigra* + *Salix alba* + *Salix atrocinerea *	1.55
*Castanea sativa* + *Fagus sylvatica* + *Quercus robur *	1.23
**High-PD**	3.24 ± 0.89
*A. glutinosa* + *S. alba* + *C. sativa*	4.79
*C. avellana* + *S. atrocinerea* + *F. sylvatica*	1.83
*B. celtiberica* + *P. nigra* + *Q. robur*	3.11

### Biodiversity effects on ecosystem functioning may depend on experimental conditions and on the biodiversity measure used

Our study supports previous evidence that plant biodiversity loss can affect litter decomposition and associated processes in stream ecosystems. However, it suggests that effects can be variable depending on the available litter and detritivore numbers used, and hence the biological interactions allowed. Experimental conditions thus seem to be main determinants of outcomes, which have been variable among different field and microcosm experiments^46^. This is particularly true for field studies, which have often found positive, negative and/or no effects at different sites^29^ or for different litter mixtures^47,48^. Many microcosm experiments have found positive diversity effects^15,28,31,49^, but these sometimes depended on which species were lost^50^, and here we found negative diversity effects. Contrasting results could be related to differences in experimental conditions, mainly regarding two aspects.

Firstly, studies or sites with more diverse detritivore assemblages have more potential for complementary resource use^29^. However, at the same time, the balance between different positive and negative interspecific and intraspecific interactions mediating diversity effects is more variable^51,52^, which may obscure the results (as discussed for microbial decomposition above). This, however, may not apply to many microcosm experiments, which use a single detritivore species, although intraspecific interactions could also play a role^53^, for example between individuals with different body size^54^, and due to density-dependent effects^55^. In our experiment, each microcosm contained 2 individuals, which differed from other experiments using more individuals per microcosm (e.g., 3^15^; 6^50^), hence with more potential for intraspecific interactions (and positive diversity effects) in the latter.

Secondly, the amount and types of litter provided could influence the results of microcosm experiments (but not so much in field studies, where litter other than that provided within litter bags is generally available in the stream). This may also help explain the outcome of our study (a negative diversity effect on decomposition) compared to other microcosm experiments. In particular, we provided litter in large excess, and > 60% of the litter preferred by detritivores (*A. glutinosa*) remained at the end of the experiment in mixtures; in contrast, others have provided more limited amounts^31^. The presence of a limiting amount of the preferred litter in mixtures may enhance the consumption of other litter types, and thus enhance overall decomposition compared to monocultures. However, this may not happen if the preferred litter is highly abundant in mixtures, because detritivores would feed mostly on it and there would be no differences with monocultures.

Another relevant question raised here is how to measure biodiversity in these studies. We found that phylogenetic distance had no effect on decomposition, but it influenced nutrient dynamics, which would have been only partially assessed by exploring species richness only. This is despite the fact that phylogenetic distance and trait variability were not strongly related, at least in relation to the traits that we measured. The inclusion of other traits such as tannins (which are generally high in the Fagaceae) would most likely have increased this relationship, so further experiments with more measured traits would be helpful to further explore the role of trait-based biodiversity measures compared to phylogenetic distance on nutrient dynamics. Moreover, our results should be taken with caution because we did not include all possible high-PD mixtures resulting from different combinations of the plant species used. Given that other studies have found either significant^27,56^ or non-significant effects^25^ of phylogenetic distance on litter decomposition in streams, and that its effects on associated processes such as nutrient dynamics are mostly unknown, we suggest that this issue merits further investigation.

## Methods

### Litter and detritivores

The plants used in the experiment were 3 species from the family Betulaceae *(Alnus glutinosa* (L.) Gaertner, *Corylus avellana* L. and *Betula celtiberica* Rothm. & Vasc*.),* 3 from the family Salicaceae *(Populus nigra* L.*, Salix alba* L. and *Salix atrocinerea* Brot.*)* and 3 from the family Fagaceae *(Castanea sativa* Mill.*, Fagus sylvatica* L. and *Quercus robur* L.). These 9 species represented common litter inputs to headwater streams in our study area. Leaves were collected from the forest floor immediately after natural abscission in the autumn of 2017 from different locations in northern Spain: *A. glutinosa, C. avellana, C. sativa* and *Q. robur* at the Agüera stream catchment (43.20°N, 3.26°W); *B. celtiberica* and *F. sylvatica* at Urkiola natural park (43.32°N, 2.97°W); *S. alba* at Mungia (43.33°N, 2.80°W); *S. atrocinera* at the Biscay campus of the University of the Basque Country (43.32°N; 2.97°W); and *P. nigra* at Barakaldo (43.29°N; 2.99°W). Leaves were cut in fragments of about 4 cm^2^ avoiding the basal midrib, air dried, and weighed to the nearest 0.01 mg using a precision balance.

Detritivores were larvae of the cased caddisfly *Sericostoma pyrenaicum*, a common invertebrate in the study area that has been often used in microcosm experiments assessing litter decomposition^34,57,58^. Detritivores were collected manually from the benthos of Perea stream (43.291°N, 3.243°W) in March 2018. The initial dry mass (DM) of experimental larvae (mean ± SE: 13.87 ± 0.56 mg) was estimated from their case length (CL, measured under a binocular microscope with an accuracy of 0.5 mm; mean ± SE: 12.51 ± 0.22 mm) and the relationship DM = 0.1398e^CL*0.2818^ (r^2^ = 0.899). This relationship was calculated using 35 additional larvae that were collected simultaneously (and with a similar case length range to experimental larvae; mean ± SE: 11.65 ± 0.52 mm), measured as above, uncased, freeze-dried and weighed. Experimental larvae were starved for 48 h just before the start of the experiment; the additional larvae were also starved for 48 h before being measured and weighed.

### Experimental setup

Litter treatments consisted of the 9 monocultures and six 3-species mixtures, either of low PD (species from the same family) or high PD (each species randomly assigned from each of the 3 families; Table 2). We explored whether high-PD mixtures had greater trait variability than low-PD mixtures using Rao’s quadratic diversity (RaoQ; dbFD function in the ‘FD’ package), which is the sum of pairwise functional distances of measured traits between species in a mixture weighted by their relative abundances^59,60^. RaoQ was higher in two high-PD mixtures than in low-PD ones, with the exception of the mixture composed by the 3 species of family Betulaceae, which had a higher value that one of the high-PD mixtures (Supplementary Table S2).

The experiment was carried out in March–April 2018 in 150 microcosms placed within a temperature-controlled room at 10 ºC (which mimicked natural conditions and minimized evaporation), with constant aeration and a light:dark regime of 12:12 h. The microcosms consisted of 580-mL glass jars (8 cm diameter, 11 cm height) containing 400 mL of stream water (Perea stream; soluble reactive P: 4.32 ± 1.25 μg P L^−1^; dissolved inorganic N: 369.55 ± 37.59 μg N L^−1^; n = 8) filtered through a 100-μm mesh (which allowed the entrance of microorganisms); and 30 cm^3^ of sediment, composed of equal parts of fine sand (200 µm–1 mm) and small gravel (0.5–1.5 cm), collected from the river bed and sterilized by incineration (550 °C, 4 h). Each microcosm received 1.5 g of air-dried litter fragments (an amount that avoided resource limitation during the experiment) belonging to 1 plant species (monocultures) or to 3 species (0.5 g per species), with 10 microcosms per litter treatment. Litter fragments of the same species were kept together using safety pins to facilitate species identification at the end of the experiment; the same was done in monocultures to avoid any possible confounding effect. Litter was incubated for 72 h (with water replacement after the first 48 h) to allow the leaching of soluble compounds and initial microbial conditioning. Water was replaced with filtered (100 μm) stream water, and 7 microcosms per treatment received detritivores (2 larvae per microcosm), while 3 microcosms per treatment remained without detritivores (in order to quantify microbial processes). We used higher replication in microcosms with detritivores because these have shown greater variability than microcosms without detritivores in previous experiments^15,34^.

During the experiment, water was replaced weekly (days 7, 14, 21, 28 and 35), and the experiment finished on day 42; on each replacement, water was filtered through a 100-μm mesh in order to avoid loss of litter fragments and detritivores. At the end of the experiment (day 42) litter was collected, sorted by species in mixtures, oven-dried, weighed to determine final DM, and then divided in 2 subsamples. One was incinerated and re-weighed to determine final ash free dry mass (AFDM); the other was used to determine final N content (using a Perkin Elmer series II CHNS/O elemental analyzer) and P content (measured spectrophotometrically after autoclave-assisted extraction^61^). From the 3 microcosms without detritivores and 3 out of the 7 microcosms with detritivores in each treatment, and before oven-drying the litter, we cut 12-mm diameter discs (5 per species) using a cork borer; discs were freeze-dried, weighed and processed in order to measure lipid ergosterol, with procedures slightly modified from Newell et al.^62^ and Suberkropp and Weyers^63^ (Supplementary Methods). Detritivores remained 48 h in starvation within the microcosms, so they were in the same conditions as at the start of the experiment; on day 44 they were uncased, freeze-dried, weighed individually to calculate their final DM, and their final N and P contents were determined as above.

Twenty-seven extra microcosms (3 per species, each containing 1.5 g of air-dried litter fragments) were used to estimate the initial (post-leaching) AFDM and several litter traits. Litter fragments were collected after 72 h, and leaf toughness was measured as the pressure required to pierce the leaf tissue using a steel rod (kPa). Then litter was oven-dried (70 °C, 72 h), weighed and divided in two subsamples. One was used to determine initial N and P contents (as above) and SLA [ratio of disc area (mm^2^) to DM (mg)]. The other was incinerated (550 °C, 4 h) and re-weighed to determine the ash content and the relationships between air-dried and oven-dried DM, and between post-leaching DM and AFDM.

### Data analyses

Survival of detritivores was 100% during the experiment but 2 larvae pupated, so those microcosms were excluded for the analyses. We calculated RaoQ for each litter mixture (see above) and for each litter trait (i.e., the variability of each particular trait in a mixture). Litter decomposition was quantified through proportional LML, calculated as the difference between initial and final AFDM divided by initial AFDM. In microcosms with detritivores, we standardized LML using mean detritivore initial DM, in order to remove any possible effects due to differences in detritivore size across microcosms. Detritivore growth was calculated as the difference between final and initial DM divided by the initial DM. We quantified nutrient dynamics through the proportional change in litter and detritivore N and P contents (i.e., the difference between final and initial N or P content divided by initial N or P content). Initial data exploration using Cleveland dot- and boxplots revealed some potential outliers (2 data points for LML, including 1 in microcosms with detritivores and 1 in microcosms without detritivores, and 4 for detritivore growth; < 5% of the data), which were removed for subsequent analyses^64^.

We explored the effect of plant SR on LML and ergosterol through the net diversity effect, which is the difference between the observed value of the response variable in a mixture and the expected value based on the values of the corresponding monocultures (net_LML_ = LML_O_ − LML_E_)^65^. Moreover, in order to explore the mechanisms driving any net diversity effect, we partitioned this net diversity effect into complementarity effects and selection effects. The complementarity effect was calculated as the average deviation from expected LML of species in a mixture multiplied by the mean LML of species in monoculture and the number of species (n) in the mixture (mean ΔLML × mean LML × n), and the selection effect was calculated as the covariance between the deviation from the expected LML of species in a mixture and their LML in monoculture, multiplied by the number of species [cov(ΔLML,LML) × n]^29,30,65^. For nutrient dynamics and detritivore growth, the existence of both positive and negative values precluded the interpretation of net diversity effects, so we directly examined differences among monocultures, low-PD and high-PD mixtures.

We ran linear mixed-effects models (lme function, ‘nlme’ package) testing for the effect of PD and detritivore presence (fixed factor fitted as an interaction) on all measured variables. Litter mixture was a random factor, and differences in variance between treatments with and without detritivores were considered using the VarIdent structure. As the interaction between PD and detritivore presence was not significant for any variable, we also ran separated models for microcosms with and without detritivores, to test for (1) differences between low-PD and high-PD mixtures for net diversity, complementarity and selection effects on LML and ergosterol; (2) differences between monocultures and low-PD and high-PD mixtures for litter N and P change; and (3) differences between monocultures and low-PD and high-PD mixtures in microcosms with detritivores for detritivore growth and detritivore N and P change. In all cases, we also calculated ordinary nonparametric bootstrapped 95% confidence intervals (BCa method using the ‘boot’ function on boot R package, based on 999 bootstrap replicates^66,67^ to determine whether the confidence intervals contained the value of zero (i.e., the null expectation of no effect or no change). All statistical analyses were performed in R statistics software^68^.

## Supplementary information

Supplementary Information 1.

## Data Availability

Data are available in the Electronic Supplementary Information.
