# Supplementary material for: Effects of two measures of riparian plant biodiversity on litter decomposition and associated processes in stream microcosms
Source: Sci Rep. 2020 Nov 12;10:19682. doi: 10.1038/s41598-020-76656-4 (PMC7661703; doi:10.1038/s41598-020-76656-4)
Supplement: Supplementary file 1 — Supplementary Information 1. [file 41598_2020_76656_MOESM1_ESM.docx]

**Effects of two measures of riparian plant biodiversity on litter decomposition and associated processes in stream microcosms**

Naiara López-Rojo, Javier Pérez, Ana Basaguren, Jesús Pozo, Juan Rubio-Ríos, J. Jesús Casas & Luz Boyero

**SUPPLEMENTARY MATERIAL**

**Supplementary Methods**

Ergosterol was extracted from the frozen-dried litter discs (≈50 mg) in 10 mL screw-cap test tubes by 30 m of refluxing in 2 mL of KOH-methanol at 80 ºC using a dry-bath system. Once cooled at room temperature, the mixture was treated with 1 mL of a saturated NaCl solution (≈ 0.36 g mL^-1^) to saturate the aqueous-phase, and sterols were extracted from the alcoholic base by partitioning with the addition of 1 mL of n-hexane (HPLC grade). Samples were then stirred in a vortex mixer for 30 s and centrifuged for 4 m at 1165RCF. The supernatant (n-hexane phase containing sterols) was collected, transferred to 1.5 mL HPLC vials, and evaporated to dryness under a stream of N_2_. A second extraction was carried out adding another 1 mL of n-hexane to the sample test tube, and repeating the above process in the same HPLC vial. The dry residue was dissolved in 1 mL of methanol (HPLC grade) and immediately injected into a high-pressure liquid chromatography system (HPLC).

**Table S1.** Litter traits (mean ± SE) measured for each plant species: nitrogen (N), phosphorus (P) and ash contents (%), specific leaf area (SLA; mm^2^ · mg^-1^) and leaf toughness (kPa).

| **Plant species** | **%N** | **%P** | **%Ash** | **SLA** | **Toughness** |
| --- | --- | --- | --- | --- | --- |
| *A. glutinosa* | 3.05 ± 0.11 | 0.082 ± 0.002 | 13.81 ± 3.33 | 17.47 ± 0.29 | 1672 ± 174 |
| *C. avellana* | 1.38 ± 0.05 | 0.069 ± 0.004 | 18.60 ± 3.13 | 19.54 ± 2.74 | 1563 ± 116 |
| *B. celtibérica* | 1.61 ± 0.13 | 0.049 ± 0.001 | 9.38 ± 1.26 | 16.41 ± 0.49 | 2271 ± 161 |
| *P. nigra* | 1.53 ± 0.09 | 0.136 ± 0.002 | 15.57 ± 0.64 | 11.90 ± 0.75 | 3859 ± 715 |
| *S. alba* | 1.80 ± 0.10 | 0.116 ± 0.002 | 23.41 ± 1.10 | 12.97 ± 0.51 | 3036 ± 116 |
| *S. atrocinerea* | 1.79 ± 0.06 | 0.092 ± 0.005 | 21.36 ± 6.27 | 13.86 ± 0.56 | 2371 ± 137 |
| *C. sativa* | 1.40 ± 0.07 | 0.054 ± 0.003 | 12.63 ± 2.06 | 19.64 ± 0.99 | 1640 ± 107 |
| *F. sylvatica* | 0.91 ± 0.12 | 0.053 ± 0.003 | 14.70 ± 4.26 | 17.87 ± 2.25 | 2142 ± 187 |
| *Q. robur* | 1.41 ± 0.13 | 0.053 ± 0.006 | 9.57 ± 1.14 | 14.71 ± 0.86 | 2752 ± 117 |

**Table S2.** Mean value and variability (RaoQ) of litter traits for each litter mixture: nitrogen (N), phosphorus (P) and ash contents (%), specific leaf area (SLA; mm^2^ · mg^-1^) and leaf toughness (kPa).

|  | | | **%N** | | **%P** | | **%Ash** | | **SLA** | | **Toughness** | |
| --- | --- | --- | --- | --- | --- | --- | --- | --- | --- | --- | --- | --- |
| **Litter mixture** | | | **mean** | **RaoQ** | **mean** | **RaoQ** | **mean** | **RaoQ** | **mean** | **RaoQ** | **mean** | **RaoQ** |
| **Low-PD** | | |  |  |  |  |  |  |  |  |  |  |
|  | | *A. glutinosa + B. celtiberica + C. avellana* | 2.00 | 1.56 | 0.07 | 0.19 | 13.90 | 0.60 | 17.80 | 0.21 | 1840 | 0.17 |
|  | | *P. nigra + S. alba + S. atrocinerea* | 1.71 | 0.04 | 0.12 | 0.32 | 20.14 | 0.47 | 12.90 | 0.08 | 3095 | 0.64 |
|  | | *C. sativa + F. sylvatica + Q. robur* | 1.24 | 0.16 | 0.05 | 0.00 | 12.28 | 0.19 | 17.34 | 0.52 | 2192 | 0.36 |
| **High-PD** | | |  |  |  |  |  |  |  |  |  |  |
|  | *A. glutinosa + S. alba + C. sativa* | | 2.06 | 1.42 | 0.08 | 0.67 | 16.60 | 0.98 | 16.72 | 0.98 | 2115 | 0.75 |
|  | *C. avellana + S. atrocinerea + F. sylvatica* | | 1.34 | 0.37 | 0.07 | 0.27 | 18.05 | 0.32 | 17.18 | 0.68 | 2023 | 0.20 |
|  | *B. celtiberica + P. nigra + Q. robur* | | 1.51 | 0.02 | 0.08 | 1.60 | 11.38 | 0.33 | 14.39 | 0.41 | 2939 | 0.74 |

**Table S3.** Results of linear mixed-effects models testing for the effect of diversity on the response variables in presence and absence of detritivores. Diversity levels were low-PD and high-PD treatments for net, complementarity and selection effects on decomposition (measured through leaf mass loss, LML) and fungal biomass (measured through ergosterol); and monocultures vs. low-PD vs. high-PD treatments for the change in litter and detritivore nitrogen (N) and phosphorus (P) and detritivore growth. df: numerator and denominator degrees of freedom; F: F-statistic value; p: p-value

|  |  | **With detritivores** | | **Without detritivores** | |
| --- | --- | --- | --- | --- | --- |
| **Variable** | **df** | **F** | **p** | **F** | **p** |
| Net LML | 1,4 | 0.222 | 0.661 | 1.639 | 0.269 |
| Complementarity LML | 1,4 | 0.310 | 0.606 | 0.006 | 0.941 |
| Selection LML | 1,4 | 0.076 | 0.795 | 0.327 | 0.597 |
| Net Ergosterol | 1,4 | 0.011 | 0.920 | 1.963 | 0.233 |
| Complementarity Ergosterol | 1,4 | 1.049 | 0.363 | 0.234 | 0.653 |
| Selection Ergosterol | 1,4 | 3.218 | 0.147 | 0.451 | 0.538 |
| Change in litter N | 2,12 | 2.222 | 0.151 | 1.451 | 0.272 |
| Change in litter P | 2,12 | 1.390 | 0.286 | 0.651 | 0.538 |
| Detritivore growth | 2,12 | 0.049 | 0.951 |  |  |
| Change in detritivore N | 2,12 | 14.538 | <0.001 |  |  |
| Change in detritivore P | 2,12 | 1.429 | 0.279 |  |  |

**Table S4**. Contribution (%) of complementarity and selection effects to the net diversity effect (sum of absolute value complementarity and selection effects) of litter mass loss (LML) and ergosterol content for each treatment (low- or high-PD) with and without detritivores.

|  | | **With detritivores** | | | **Without detritivores** | |
| --- | --- | --- | --- | --- | --- | --- |
| **Variable** | | **Complementarity** | | **Selection** | **Complementarity** | **Selection** |
| LML | | |  |  |  |  |
|  | Low PD | | 85.66 | 14.34 | 60.38 | 39.62 |
|  | High PD | | 88.47 | 11.53 | 45.40 | 54.60 |
| Ergosterol | | |  |  |  |  |
|  | Low PD | | 91.04 | 8.96 | 83.01 | 16.99 |
|  | High PD | | 81.68 | 18.32 | 53.35 | 46.65 |
